# Central and peripheral pathology of kuru: pathological analysis of a recent case and comparison with other forms of human prion disease

**DOI:** 10.1098/rstb.2008.0091

**Published:** 2008-11-27

**Authors:** Sebastian Brandner, Jerome Whitfield, Ken Boone, Anderson Puwa, Catherine O'Malley, Jacqueline M. Linehan, Susan Joiner, Francesco Scaravilli, Ian Calder, Michael P. Alpers, Jonathan D.F. Wadsworth, John Collinge

**Affiliations:** 1 MRC Prion Unit and Department of Neurodegenerative Disease, UCL Institute of Neurology, National Hospital for Neurology and NeurosurgeryQueen Square, London WC1N 3BG, UK; 2 Papua New Guinea Institute of Medical ResearchPO Box 60, Goroka, EHP 441, Papua New Guinea; 3 Centre for International Health, ABCRC, Shenton Park Campus, Curtin UniversityGPO Box U1987, Perth, WA 6845, Australia

**Keywords:** kuru, Creutzfeldt–Jakob disease, neuropathology

## Abstract

While the neuropathology of kuru is well defined, there are few data concerning the distribution of disease-related prion protein in peripheral tissues. Here we report the investigation of brain and peripheral tissues from a kuru patient who died in 2003. Neuropathological findings were compared with those seen in classical (sporadic and iatrogenic) Creutzfeldt–Jakob disease (CJD) and variant CJD (vCJD). The neuropathological findings of the kuru patient showed all the stereotypical changes that define kuru, with the occurrence of prominent PrP plaques throughout the brain. Lymphoreticular tissue showed no evidence of prion colonization, suggesting that the peripheral pathogenesis of kuru is similar to that seen in classical CJD rather than vCJD. These findings now strongly suggest that the characteristic peripheral pathogenesis of vCJD is determined by prion strain type alone rather than route of infection.

## 1. Introduction

Prion diseases are fatal neurodegenerative disorders that include Creutzfeldt–Jakob disease (CJD), Gerstmann–Sträussler–Scheinker disease, fatal familial insomnia, kuru and variant CJD (vCJD) in humans (Collinge 2005; Wadsworth & Collinge 2007). Their central feature is the post-translational conversion of host-encoded, cellular prion protein (PrP^C^) to an abnormal isoform, designated PrP^Sc^ (Prusiner 1982; Collinge 2001; Collinge & Clarke 2007). Human prion diseases are biologically unique in that the disease process can be triggered through inherited germ line mutations in the human prion protein gene (*PRNP*), infection (by inoculation, or in some cases by dietary exposure) with prion-infected tissue or by rare sporadic events that generate PrP^Sc^ (Prusiner 1998; Collinge 2001, 2005; Weissmann 2004; Wadsworth & Collinge 2007). Substantial evidence indicates that an abnormal PrP isoform is the principal, if not the sole, component of the transmissible infectious agent, or prion (Prusiner 1998; Collinge 2001; Weissmann 2004; Collinge & Clarke 2007).

Kuru provides our principal experience of an epidemic human prion disease and affected the Fore linguistic group of the Eastern Highlands of Papua New Guinea and to a lesser extent neighbouring groups with whom the Fore intermarried (Zigas & Gajdusek 1959; Alpers 1987; Collinge & Palmer 1997; Mead *et al*. 2003; Collinge *et al*. 2006). It was the practice in these communities to engage in consumption of dead relatives as a mark of respect and mourning (transumption). Kuru was the first human prion disease shown to be transmissible, by inoculation of non-human primates with autopsy-derived brain tissue (Gajdusek *et al*. 1966).

Consistent with the hypothesis that kuru originated from chance consumption of an individual with sporadic CJD (sCJD; Alpers & Rail 1971), molecular and biological strain typing studies have shown that kuru prions have molecular strain types (Parchi *et al*. 1997, 2000; Wadsworth *et al*. 2008*a*) and transmission properties (Brown *et al*. 1994; Wadsworth *et al*. 2008*a*) equivalent to those of classical (sporadic and iatrogenic) CJD prions rather than vCJD prions or inherited forms of prion disease (Wadsworth *et al*. 2008*c*). Despite these data, both the clinical presentation and the neuropathology of kuru are distinct from the majority of patients with sCJD. In contrast to a rapidly progressive dementia that is seen in most cases of sCJD (Brown *et al*. 1987; Parchi *et al*. 1996, 1999; Collinge 2001, 2005; Hill *et al*. 2003; Wadsworth *et al*. 2003; Collins *et al*. 2006), kuru presents with progressive cerebellar ataxia with dementia appearing as a later and less prominent feature (Alpers 1987; Brown *et al*. 1994; Collinge & Palmer 1997; Collinge 2005; Collinge *et al*. 2006; although see Collinge *et al*. (2008) for clinical review of recent cases). Moreover, although the neuropathological changes seen in kuru lie within the spectrum of those seen in sCJD, unicentric PrP plaques are unusually prominent and widespread (Hainfellner *et al*. 1997; McLean *et al*. 1998). As a progressive cerebellar syndrome and the occurrence of kuru-type plaques reminiscent of kuru are also notable features of iatrogenic CJD resulting from peripheral exposure to sCJD prions (Brown *et al*. 1992, 2000, 2006; Billette de Villemeur *et al*. 1994; Will 2003), it appears that the cerebellar onset and subsequent neuropathological changes in kuru may be significantly determined by peripheral routes of infection (predominantly dietary), rather than by prion strain type (Wadsworth *et al*. 2008*a*).

Although the pathological consequences of prion infection occur in the central nervous system (CNS) and experimental transmission of these diseases is most efficiently accomplished by intracerebral inoculation, natural infections do not occur by these means. Administration to sites other than the CNS is known to be associated with much longer incubation periods (Brown *et al*. 2000; Collinge 2001), and kuru demonstrates that human prion disease incubation periods may extend to 50 years or more (Collinge *et al*. 2006). Experimental evidence suggests that this latent period is associated with clinically silent prion replication in lymphoreticular or other tissues, whereas neuroinvasion takes place later (Kimberlin & Walker 1988; Fraser *et al*. 1992; Aguzzi 2003). Distinct forms of prion disease show differences in lymphoreticular involvement that may be related to the aetiology of the disease or to the divergent properties of distinct prion strains (Collinge & Clarke 2007). For example, the tissue distribution of PrP^Sc^ in vCJD differs strikingly from that in classical CJD or inherited prion disease with uniform and prominent involvement of lymphoreticular tissues (Hill *et al*. 1999, 2006; Wadsworth *et al*. 2001; Glatzel *et al*. 2003; Head *et al*. 2004; Hilton *et al*. 2004; Joiner *et al*. 2005; Wroe *et al*. 2006). The extensive peripheral pathogenesis seen in vCJD raises concerns that iatrogenic transmission of vCJD prions may be a major public health issue (Collinge 1999, 2001, 2005; Wadsworth & Collinge 2007) and disturbingly, cases of blood transfusion-associated vCJD prion infection have now emerged (Llewelyn *et al*. 2004; Peden *et al*. 2004; Wroe *et al*. 2006).

To date, although the neuropathology of kuru is well defined (Fowler & Robertson 1959; Klatzo *et al*. 1959; Neumann *et al*. 1964; Beck & Daniel 1965; Kakulas *et al*. 1967; Hainfellner *et al*. 1997; Lantos *et al*. 1997; McLean *et al*. 1998), there are few data concerning the distribution of abnormal PrP deposition or prion infectivity in peripheral tissues in kuru (Brown *et al*. 1994). Here, we now describe the investigation of disease-related PrP in both the brain and the lymphoreticular tissues of a kuru patient who died in 2003.

## 2. Material and methods

### (a) Research governance

Collection, storage and analysis of human tissue samples were performed with consent from relatives and local community leaders. Ethical approval for these studies was obtained from the Local Research Ethics Committee of UCL Institute of Neurology/National Hospital for Neurology and Neurosurgery and the Medical Research Advisory Committee of the Government of Papua New Guinea.

### (b) Immunohistochemistry

Brain and peripheral tissues were analysed with anti-glial fibrillary acidic protein (GFAP), rabbit polyclonal antiserum and anti-PrP monoclonal antibody ICSM 35 (D-Gen Ltd, London, UK), using a Ventana automated immunohistochemical staining machine (Ventana Medical Systems, Inc., Tucson, AZ), as described previously (Frosh *et al*. 2004; Wadsworth *et al*. 2008*b*). Tissue was fixed in 10% buffered formol saline followed by incubation in 98% formic acid for 1 hour. Following postfixation for 24 hours in 10% buffered formol saline, the tissue samples were processed through graded alcohols and paraffin wax embedded. The sections were cut at a nominal thickness of 4 μm, treated with 98% formic acid for 5 min and then boiled in a low ionic strength buffer (2.1 mM Tris, 1.3 mM EDTA, 1.1 mM sodium citrate, pH 7.8) for 20 min. Abnormal PrP accumulation was examined using anti-PrP monoclonal ICSM 35 (D-Gen Ltd) followed by a biotinylated anti-mouse IgG secondary antibody (*i*View Biotinylated Ig, Ventana Medical Systems, Inc.) and an avidin–biotin horseradish peroxidase conjugate (*i*View SA-HRP, Ventana Medical Systems, Inc.) before development with 3′,3-diaminobenzedine tetrachloride as the chromogen (*i*View DAB, Ventana Medical Systems Inc.). Haematoxylin was used as the counter stain. Haematoxylin and eosin (H&E) staining of serial sections was performed using conventional methods. Appropriate controls were used throughout.

### (c) Immunoblotting

All procedures were carried out in a microbiological containment level 3 facility, with strict adherence to safety protocols. Brain (frontal cortex) and peripheral tissues were prepared as 10% w/v homogenates in Dulbecco's sterile phosphate buffered saline lacking Ca^2+^ and Mg^2+^ ions using Duall tissue grinders (Wadsworth *et al*. 2001, 2008*b*). Brain homogenate was analysed, before or after proteinase K digestion (50 μg ml^−1^ final protease concentration, 1 hour, 37°C), by immunoblotting with anti-PrP monoclonal antibody 3F4 using high sensitivity enhanced chemiluminescence (Wadsworth *et al*. 2001, 2008*b*). Peripheral tissue homogenate was analysed by sodium phosphotungstic acid precipitation of PrP^Sc^, proteinase K digestion and immunoblotting with anti-PrP monoclonal antibody 3F4 using high sensitivity enhanced chemiluminescence, as described previously (Wadsworth *et al*. 2001, 2008*b*).

## 3. Results

### (a) Summary of clinical history

Detailed clinical description is given in Collinge *et al*. (2008; patient KAW), but is also outlined here. The patient was male and aged 58 years when examined in September 2001. He had experienced episodes of pain and weakness in the legs for several years, which had made walking difficult; these had initially responded to local treatment but then recurred. He also had headaches and pain in the neck, arms and thoracic and abdominal muscles. He was convinced these were ‘attacks of kuru’. Frequent fasciculations in his calf muscles, a steady posture, firm stance and normal gait were noted. He was born in 1943 and lived in the South Fore continuously during the period when traditional mortuary feasts were held. His mother died of kuru in 1965 and local oral history confirmed his participation in multiple mortuary feasts as a child.

On his return from a month of travelling in the highlands, he complained of unsteadiness of gait, which slowly worsened. Over the next 10 months, he followed a progressive course with worsening cerebellar ataxia typical of kuru and by the end of August 2002 had entered the second (sedentary) stage. After three months, he was unable to sit without support and became recumbent (stage 3 of kuru). He alternated between periods of confusion and lucidity. He was examined approximately a month after he became recumbent when he was well nourished and in no pain. He was lucid and able to converse sensibly. He was calm and rational, with some flattening of affect. He had insight into his disease and said that his only worry was what would happen to his children after he died. He was continent of urine and faeces and without pressure sores. He had severe photophobia and pronounced cerebellar dysarthria. Eye movements were full but jerky, with no nystagmus; there were no abnormal facial movements or dysconjugate eye movements. He was able to sit up only with external support and had marked truncal instability. His hands were held clasped in front to suppress involuntary postural tremors. He had plastic, jerky rigidity of all four limbs. His legs were weak and power in the arms and hands was also reduced to grade 3 out of 5. Tendon reflexes were diminished or absent; plantar responses were flexor. There was dysmetria in finger and hand movements, intention tremor in finger-nose alternation and dysdiadochokinesis. He performed all these tests without hesitation or confusion.

His physical condition progressively declined and he became incontinent, developed pressure sores in his sacral area, in both buttocks and on both heels, and dislocated his left hip. He ate very little and subsisted largely on sips of water. He was unable to speak but continued to make eye contact and followed people with his eyes. He persisted in this state for another four months. In April 2003, he lost consciousness for a day but by the next day had regained the visual awareness of his surroundings and again made eye contact; the only movement was of his eyes. He remained in this state for 3 days. He was totally moribund the next day and died the following day. *PRNP* analysis demonstrated normal coding sequence and the codon 129 genotype was M/V.

### (b) Tissue collection and macroscopic findings

Autopsy was performed locally. After opening the skull, the brain was removed, and the brain stem and the cerebellum were separated from the forebrain at midbrain level. The forebrain was then divided sagittally. The left hemisphere was divided further and representative samples were frozen. The right forebrain and a small portion of the cerebellar hemisphere were fixed in formalin. A large number of somatic organs including lymphoreticular tissue were also collected, either frozen or formalin fixed. All tissue samples were shipped to the MRC Prion Unit, UCL Institute of Neurology.

**Figure 1:**
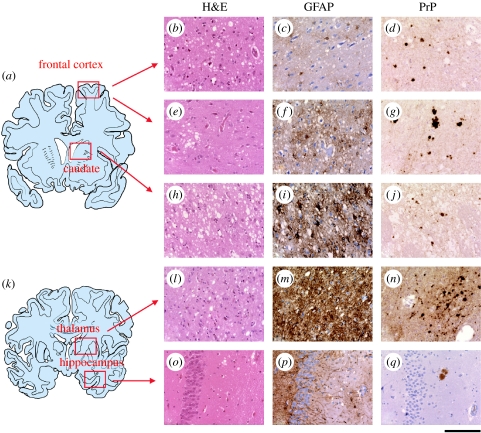
Pathological changes in several kuru forebrain regions. Multiple areas were examined and exhibited a highly variable degree of spongiform changes, gliosis and intensity and type of PrP deposition. (*a*) Sampling of frontal cortex and caudate region in a coronal brain slice, anterior third of the brain slice. (*b–d*) and (*e–g*) Two cortical areas with variable degree of spongiform degeneration, and moderate gliosis. As with most other cortical areas, there is a variable degree of synaptic PrP deposition and small dense plaques. (*a*,*h–j*) Caudate nucleus and (*k–n*) thalamus show a more significant spongiform degeneration (*h*,*l*) with more severe neuronal loss. This is accompanied by severe astrocytic reaction (gliosis; *i*,*m*). As in the cortex, there is a synaptic PrP deposition and a variable PrP plaque load (*j*,*n*). The hippocampus (*k*,*o*–*q*) is relatively mildly affected. There is almost no spongiform change (*o*) and there are only very occasional dense PrP plaques with no synaptic PrP deposition (*q*). There is, however, some astrocytic reaction (*p*). Scale bar, 120 μm.

The formalin-fixed hemisphere excluding the brainstem and the cerebellum weighed 430 g. The cerebral cortex appeared normally configured, and no other abnormalities were seen. The brain was sectioned in coronal sections of approximately 1 cm thickness, starting at the level of the mammillary bodies. The coronal slices showed a cortical ribbon of normal width, well demarcated from the underlying white matter which appeared normal, and although no apparent atrophy was seen, the brain appeared generally small. The caudate, putamen, globus pallidus, thalamus and subthalamic nucleus, and the amygdala as well as the hippocampus appeared macroscopically normal. The ventricular system was of normal appearance, with indication of hydrocephalus *ex vacuo*. Pons, medulla, cerebellar cortex and white matter and the dentate nucleus were not available for examination, but a section of inferior brainstem including olives was available and appeared normal. The small sample of the cerebellar hemisphere (25 g) did not show macroscopic abnormalities. Representative brain regions were sampled, including several neocortical areas (frontal, temporoparietal and occipital cortex; figure 1*a–g*), hippocampus (figure 1*k*,*o–q*), deep grey matter structures (caudate nucleus (figure 1*a*,*h–j*) and thalamus on two levels (figure 1*k–n*)), cerebellum (figure 3*a–d*), brain stem (level of olives; figure 3*e–h*) and spinal cord (cervical and lumbar; figure 3*i–l*).

## 4. Neuropathological findings

### (a) Frontal cortex

Two adjacent regions of the frontal cortex were examined (area rostral to the tip of the temporal lobe): spongiform degeneration was generally mild to moderate, with fine vacuolation (figure 1*b*), which was occasionally confluent (figure 1*e*). In the H&E stained sections, occasional amyloid plaques were identified. Gliosis (assessed by immunohistochemical stainings using anti-GFAP antiserum; figure 1*c*,*f*) was brisk and showed diffuse fibrillary gliosis in the white matter and individual stellate astrocytes in the grey matter. Abnormal PrP was present as synaptic deposits when present at low density and at higher density formed dense plaques, which varied in size, ranging from small dense granules to plaques of up to 30 μm in diameter (figure 1*d*,*g*).

**Figure 2:**
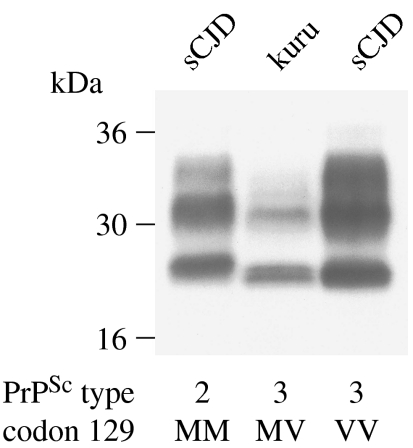
Frontal cortex homogenates from the kuru patient and sCJD control samples of known PrP^Sc^ type were digested with proteinase K and analysed by enhanced chemiluminescence using anti-PrP monoclonal antibody 3F4. The provenance of each brain sample is designated above each lane and the type of PrP^Sc^ detected in each sample (using the London classification of human PrP^Sc^ types; Collinge *et al*. 1996; Hill *et al*. 2003) and the *PRNP* codon 129 genotype of the patient (M, methionine; V, valine) are designated below.

Analysis of frontal cortex brain homogenate by immunoblotting after limited proteinase K digestion demonstrated the presence of type 3 PrP^Sc^ (figure 2), which we have previously observed in sporadic and iatrogenic CJD (Collinge *et al*. 1996; Wadsworth *et al*. 1999; Hill *et al*. 2003) and in kuru (Wadsworth *et al*. 2008*a*,*c*).

### (b) Caudate nucleus

The caudate nucleus was examined at two levels (level of the nucleus accumbens and level of the anterior commissure). Here, spongiform degeneration was far more intense and severe, with larger vacuoles, which were often confluent, indicating widespread neuronal death (figure 1*h*). This spongiform degeneration was seen across the entire grey matter of the caudate nucleus. In correlation with the severe degeneration, gliosis was more substantial with brisk astrocytic reaction (figure 1*i*). PrP deposits were mainly synaptic with occasional formation of small plaques. The white matter structures contained only fine granular deposits of abnormal PrP (figure 1*j*).

### (c) Thalamus

Several thalamic nuclei (level of subthalamic nucleus and pulvinar) showed a moderate spongiosis but a more severe neuronal loss than the caudate nucleus (figure 1*l*). This led to a compaction of the grey matter, with surviving astrocytes being the main population. This is reflected by a very dense fibrillary gliosis throughout (figure 1*m*). Abnormal PrP accumulated as dense synaptic deposits, which often became confluent to form dense plaque-like structures (figure 1*n*). These were most accentuated in areas of severe neuronal loss and spongiform degeneration. However, large classical kuru-type PrP plaques were not seen (figure 1*n*).

### (d) Hippocampus

The hippocampus was examined at the level of the lateral geniculate nucleus, showing the ribbon of the dentate gyrus and Ammon's horn, extending to the entorhinal cortex. Generally, the entire region was spared by the various pathological changes seen elsewhere in the brain. Occasional spongiform vacuoles were seen in the stratum radiatum and in the hilus (figure 1*o*), and some mild spongiosis in the entorhinal cortex. There was no neurodegeneration in the dentate gyrus and Ammon's horn (figure 1*o*). Accordingly, there was relatively little gliosis, this being restricted mainly to the stratum radiatum (figure 1*p*). PrP was deposited as occasional small circumscribed plaques, but there was almost no detectable synaptic PrP (figure 1*q*).

### (e) Cerebellum

**Figure 3:**
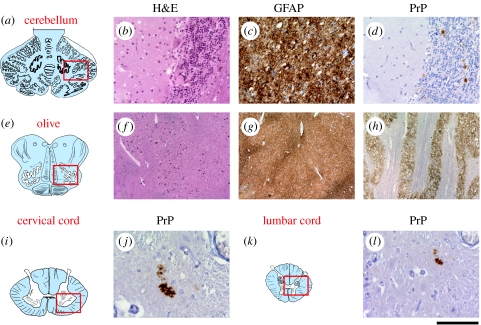
Pathological changes in the kuru cerebellum, brain stem and spinal cord. The cerebellum (*a*) shows very little spongiosis (mainly in the molecular layer (*b*)) and only occasional kuru plaques (*d*). There is a generalized strong fibrillary gliosis in the white matter (*c*) of the Bergmann glia and, to a lesser extent, in the molecular layer. Deep grey matter structures of the cerebellum (dentate gyrus, not shown) and the olives (*f–h*) show a remarkably intense deposition of synaptic PrP without the presence of plaques (*h*). This is accompanied by strong fibrillary gliosis (*g*). In the spinal cord (cervical (*i*) and lumbar segments (*k*)), there are only very occasional plaques in the grey matter (*j*,*l*). Scale bar, 120 μm (*b–d*,*j*,*l*) and 500 μm (*f–h*).

One section of the cerebellum, including the dentate nucleus, was analysed. Here, the most significant pathological changes were seen in the molecular layer, extending to the Purkinje cell layer, but not in the internal granular layer (figure 3*a–d*). Amyloid plaques were occasionally seen in the internal granular layer (figure 1*b*). Gliosis was dense fibrillary in the white matter and less pronounced in the molecular layer (figure 3*c*). Only occasional kuru plaques were seen in the internal granular layer, between Purkinje cells and in the molecular layer (figure 3*d*).

### (f) Brain stem

A section of the brain stem at the level of the olive was available for analysis. There was remarkably little spongiosis (figure 3*f*); however, a dense fibrillary gliosis was seen throughout the white and grey matter structures (figure 3*g*). Abnormal PrP was seen predominantly as synaptic deposits in the grey matter structures, such as the olivary nucleus. Small (micro) PrP plaques were seen in both the grey and white matter (figure 3*h*).

### (g) Spinal cord

We examined the spinal cord at cervical (figure 3*i*,*j*), thoracic (not shown) and lumbar levels (figure 3*k*,*l*). There was virtually no pathological abnormality detectable on the H&E stained sections, but staining for abnormal PrP revealed occasional small plaques in the grey matter of the anterior (figure 3*i*,*j*) or posterior horn (figure 3*k*,*l*).

### (h) Spinal roots

We examined spinal nerve roots for the presence of abnormal PrP and found minute PrP deposits associated with axons, possibly representing intra-axonal PrP (not shown).

### (i) Comparison of PrP plaque morphology in kuru and other human prion diseases

**Figure 4:**
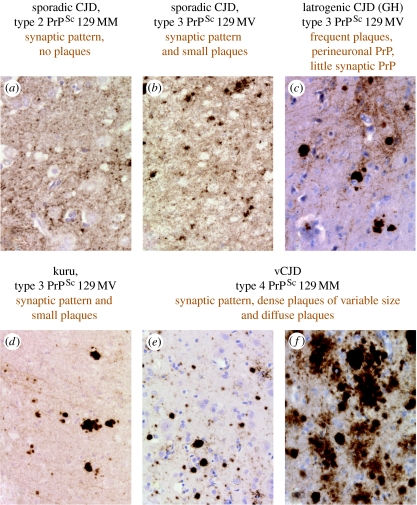
Comparison of abnormal PrP deposition in sporadic, iatrogenic and vCJD. The disease aetiology, *PRNP* codon 129 genotype of the patient (M, methionine; V, valine) and the type of PrP^Sc^ detected in each sample (using the London classification of human PrP^Sc^ types; Collinge *et al*. 1996; Hill *et al*. 2003) are designated above each brain sample. Sporadic CJD with type 2 PrP^Sc^ 129 MM typically shows a diffuse, synaptic pattern of abnormal PrP deposition (*a*), while sCJD with type 3 PrP^Sc^ 129 MV often forms small dense plaques (*b*). Propagation of type 3 PrP^Sc^ in iatrogenic CJD (caused by administration of contaminated growth hormone, GH; *c*) and kuru (*d*) show not only variably diffuse PrP deposition but also striking formation of PrP plaques in various areas of the brain. These PrP plaques are consistently distinct from those seen in vCJD (*e*,*f*), where PrP plaques are often surrounded by conspicuous vacuolation, designated ‘florid plaques’.

Figure 4 shows comparison of the typical PrP plaque pattern seen in the present kuru patient with cases of sCJD, iatrogenic CJD and vCJD. Propagation of type 3 PrP^Sc^ 129 MV in sCJD, iatrogenic CJD and kuru share fundamental histopathological patterns, showing a propensity of PrP to aggregate into plaques of variable size, ranging from small granules to medium-sized solid plaques (figure 4*b–d*). There was no particular accentuation around these plaques, and they were distinct from the florid PrP plaques (figure 4*e*,*f*) that are the neuropathological hallmark of vCJD (Will *et al*. 1996; McLean *et al*. 1998; Ironside & Head 2004).

### (j) Summary of CNS pathology

In general, there was very little pathological change in the white matter, while the grey matter showed more intense spongiosis and PrP deposition, which varied remarkably between different brain regions. This is best illustrated by the marked contrast shown in figure 3*h* which shows adjacent brain stem white matter unstained and the grey matter of the olive containing abnormal PrP. Cortical areas (figure 1*a–g*), hippocampus (figure 1*o–q*), cerebellum (figure 3*a–d*) and spinal cord (figure 3*i–l*) were least affected, in that there was very little or no spongiform change and often very discrete PrP deposition. The most severely affected structures were caudate nucleus and thalamus (figure 1*a*,*h–j*,*k–n*), which were severely devastated with massive spongiosis and neuronal loss, accompanied by a very severe astrocytic reaction and both synaptic and plaque deposition of PrP. The thalamus showed the heaviest deposition of synaptic PrP. Other grey matter structures such as the cerebellar dentate nucleus and the olive in the brain stem showed little spongiosis, but a very strong gliosis and a diffuse and intense deposition of PrP (figure 3*e–h*). In stark contrast, cerebellar cortex, hippocampus and spinal cord were almost unaffected by spongiosis or PrP deposition. There were only occasional small dense kuru plaques.

### (k) Investigation of peripheral tissues

All peripheral tissues examined by immunohistochemistry comprising heart, pericardium, lung, muscle, thymus, dura and cranial nerves 1–12 failed to show detectable amounts of abnormal PrP deposition. To investigate the lymphoreticular pathogenesis, 10% w/v homogenates of spleen and distal ileum were investigated by sodium phosphotungstic acid precipitation of PrP^Sc^ (Safar *et al*. 1998; Wadsworth *et al*. 2001). This method facilitates highly efficient recovery and detection of PrP^Sc^ from human tissue homogenate when present at levels 10^4^–10^5^-fold lower than found in brain (Wadsworth *et al*. 2001, 2007; Frosh *et al*. 2004). Nevertheless, despite analysis of several preparations of tissue homogenate by this procedure, no detectable PrP^Sc^ was found in spleen or distal ileum.

## 5. Discussion

In the present study, we have investigated the distribution of disease-related PrP in the CNS and in peripheral tissue from a kuru patient who died in 2003. Despite the unusually long incubation period in this case, the neuropathological findings faithfully reproduced the central features described previously in kuru (Fowler & Robertson 1959; Klatzo *et al*. 1959; Neumann *et al*. 1964; Beck & Daniel 1965; Kakulas *et al*. 1967; Hainfellner *et al*. 1997; Lantos *et al*. 1997; McLean *et al*. 1998). Kuru shows neuropathological changes that lie within the spectrum of those seen in sCJD but is defined by unusually prominent and widespread unicentric PrP plaques. Kuru most closely resembles iatrogenic CJD caused by peripheral administration of contaminated growth hormone (Brown *et al*. 1992, 2000, 2006; Billette de Villemeur *et al*. 1994; Will 2003) and a rare subtype of sCJD associated with long clinical duration, progressive ataxia and *PRNP* codon 129 heterozygosity (Parchi *et al*. 1996, 1999; Hill *et al*. 2003).

Recently, we reported that kuru prions have molecular and biological properties equivalent to those of classical CJD prions rather than vCJD prions (Wadsworth *et al*. 2008*a*). These findings support previous molecular (Collinge *et al*. 1996; Parchi *et al*. 1997, 2000; Wadsworth *et al*. 1999; Hill *et al*. 2003, 2006), neuropathological (Will *et al*. 1996; McLean *et al*. 1998; Ironside & Head 2004) and transmission (Bruce *et al*. 1997; Hill *et al*. 1997; Asante *et al*. 2002; Wadsworth *et al*. 2004; Bishop *et al*. 2006; Beringue *et al*. 2008) studies indicating that vCJD is a highly distinct human prion strain. The pathogenesis of vCJD differs significantly from that of other forms of human prion disease. PrP^Sc^ is readily detectable in lymphoreticular tissues in vCJD and not in classical CJD or inherited prion disease (Hill *et al*. 1999, 2006; Wadsworth *et al*. 2001; Glatzel *et al*. 2003; Head *et al*. 2004; Hilton *et al*. 2004; Joiner *et al*. 2005; Wroe *et al*. 2006). Here we now report no evidence for prion colonization of lymphoreticular tissues in kuru, indicating that the peripheral pathogenesis of kuru is also closely similar to classical CJD rather than vCJD. This is in accordance with the negative tonsil biopsy reported in an earlier kuru patient (Collinge *et al*. 2008).

Because kuru, iatrogenic CJD and vCJD are caused by a peripheral route of exposure to infectious prions, it has been speculated that extensive lymphoreticular pathogenesis may result from this common route of exposure. However, the fact that prominent lymphoreticular infection has not been detected in iatrogenic CJD (Hill *et al*. 1999; Head *et al*. 2004) or kuru (this study) contradicts this hypothesis and indicates that characteristic peripheral pathogenesis of vCJD is defined by prion strain type alone rather than route of infection.
